# Supplementary material for: G-Protein-Coupled Estrogen Receptor Expression in Rat Uterine Artery Is Increased by Pregnancy and Induces Dilation in a Ca2+ and ERK1/2 Dependent Manner
Source: Int J Mol Sci. 2022 May 26;23(11):5996. doi: 10.3390/ijms23115996 (PMC9180712; doi:10.3390/ijms23115996)
Supplement: Supplementary file 1 [file ijms-23-05996-s001.zip › ijms-1721701-supplementary.pdf]

Supplementary Materials

Table S1. Summary of molecular mechanisms and effects associated with GPER activation in the vasculature.

Specie	Performed method/ Locus	Major findings	Ref
**Human**	EA.hy926 cells/ umbilical vein	GPER activation upregulates eNOS expression via Kruppel-like factor 2-dependent pathways and increases intracellular Ca2+ levels	[60]
	Vascular smooth muscle cells (VSMCs)	Oxidized low-density lipoprotein (ox-LDL)-mediated proliferation and migration of VSMCs upregulates GPER expression	[61]
	Aortic endothelial cells	*In vitro* kaempferol-mediated activation of GPER has protective effects on postmenopausal atherosclerosis	[62]
	Brain vascular pericytes	Confirmed GPER expression	[63]
	Aortic smooth muscle cells	Aging increases vascular oxidative stress, Nox1 abundance, mobilization of intracellular calcium; promotes Ang II–induced hypertension and vascular dysfunction via GPER	[64]
	Coronary artery smooth muscle cells	GPER activation suppresses cell synthetic phenotype and proliferation via inhibition of ERK1/2 and Akt phosphorylation	[65]
	Coronary artery endothelial cells	Estrogen-inhibited LDL transcytosis occurs via GPER; higher expression of GPER vs estrogen receptors α/β	[66]
	Aortic smooth muscle cells	Bisphenol A increases cell proliferation and upregulates expression of AngII and cytokines via GPER	[67]
	Ambulatory blood pressure	Hypofunctional missense genetic variant of GPER, P16L GPER, is associated with increased blood pressure in females	[68]
	Subcutaneous fat tissue resistance artery	GPER activation induces vasodilation (higher effect in post-menopausal women compared to men and pregnant women) through eNOS/NO-signalling pathway	[69]
	Varicose vein	Increased GPER expression associated with severity of chronic venous disease	[70]
	TIVE endothelial cells	Estrogen inhibits endothelial prostanoid production via GPER under pro-inflammatory conditions	[71]
	HUVEC		[72]

	Wire myography/<br>Internal mammary artery	Protocatechuic aldehyde increases GPER expression and reduces ROS production and inflammatory markers through GPER activation<br><br>G1 causes more potent vasodilation than 17β-estradiol	[43]
**Mouse**	Aorta	*In vivo* kaempferol-mediated activation of GPER upregulates PI3K/AKT/Nrf2 pathway	[3]
	Wire myography/<br>Renal artery	Activation of GPER mediates age-dependent, Nox-mediated reduced NO/endothelium-dependent vasodilation	[73]
	Bilateral Common Stenosis/<br>Carotid artery	Increased expression of GPER by ginsenoside Rg1 improves vascular dementia	[74]
	Pressure myography/<br>Carotid artery	GPER–/– increases contractility to endothelin-1 (ET-1) and reduces ET-1-stimulated smooth muscle cell intracellular Ca2+ levels	[45]
	Wire myography/<br>Carotid artery	Estrogen inhibits prostanoid-mediated vasoconstriction via GPER under pro-inflammatory conditions	[12]
	Wire myography/<br>Mesenteric resistance artery	Aldosterone increases phenylephrine-mediated contraction in wild-type mice and ERK1/2 phosphorylation via GPER activation; inhibition of GPER contributes to reverse vascular dysfunction in db/db mice; GPER expression is increased in db/db mice	[75]
	Telemetry, wire myography/<br>Mesenteric artery	GPER–/– increases pulse pressure in Ang II-induced hypertension female mice	[76]
	Laser Doppler flowmetry/<br>Plantar artery	Cooling induces vasoconstrictor effect through GPER in skin	[77]
**Rat**	Tail-cuff device/<br>Tail artery	GPER–/– increases sensitivity to stress-induced sympathoexcitation	[78]
	Perfusion/<br>Superior mesenteric artery	Anthocyanin-mediated activation of GPER elicits NO/endothelium-dependent vasodilation	[79]
	Organ bath/<br>Lower thoracic aorta	GPER activation attenuates aortic remodelling, oxidative stress and glycosaminoglycans; elicits endothelium-independent vasorelaxation; potential for protection from salt- and pressure-induced vascular damage	[80]
	Langendorff/<br>Coronary artery	GPER modulates vascular tone; elicits higher vasodilation in female compared to male rats	[32]
	Aortic endothelial cells	Estrogen-mediated GPER-dependent ERK activation and apoptosis occurs when ERα is downregulated	[81]

	Wire myography/ Mesenteric artery	GPER mediates vasorelaxation via PI3K-Akt-eNOS and potassium channels in both sexes; higher GPER expression in males	[30, 35]
	Telemetry	Systemic GPER activation ameliorates ovariectomy-induced increase in mean arterial pressure	[82]
	Doppler ultrasound	GPER activation reverses pulmonary hypertension-induced cardiac dysfunction, right ventricle hypertrophy/collagen deposition and improves cardiac output	[83]
	Pressure myography/Renal interlobular artery	GPER activation restores contraction rate and systolic velocity after ischemia-reperfusion injury through increased eNOS expression and NO production	[84]
	Organ bath/ Aorta endothelial cells; smooth muscle cells	GPER mediates endothelium-dependent rapid vasodilatory effects of aldosterone and regulates smooth muscle cell apoptosis via activation of PI3K or inhibition of PKA ERK-dependent pathway	[85, 86]
	Aortic smooth muscle cells	Hypofunctional missense genetic variant of GPER, P16L GPER, decreases GPER-mediated ERK phosphorylation	[9]
	Perfusion/ Renal artery	In conditions of basal tone, GPER activation evokes vasoconstriction via ROCK, PKC, p38 MAPK, p42/44 MAPK, tyrosine kinase, EGF receptor kinase and VOCC mechanisms; GPER induces vasodilation when phenylephrine-elevates perfusion pressure	[87]
	Organ bath/ Aorta	GPER activation induces endothelium-dependent vasorelaxation through Src-mediated activation of the EGF receptor and the Akt pathway	[33]
	Wire myography/ Mesenteric artery	GPER induces relaxation is endothelium-dependent in females; is potentiated by cyclooxygenase inhibition in males; is NO-dependent and involves participation of H2O2 in both sexes	[88]
	Wire myography/ Mesenteric artery	GPER-dependent vasorelaxation involves endothelial NO which activates guanylyl cyclase and smooth muscle cell activation of adenylyl cyclase	[89]
	Wire myography/ Uterine artery	GPER induces endothelium-dependent vasodilation via NO/cGMP pathway; vasodilation is potentiated in pregnancy	[27]
	Wire myography/ Thoracic aorta, carotid, mesenteric and renal arteries	Greater GPER expression in the aorta of pregnant vs other vessels or non-pregnant rats; G1-induced relaxation is more potent in the aorta and mesenteric artery; aorta maximum relaxation is reduced by NOS,	[34]

	Pressure Myography/ Middle cerebral artery, smooth muscle cells	COX, hyperpolarization inhibitors and endothelial denudation<br><br>G1 elicits endothelium-independent vasodilation and reduces BK channel activation and currents	[31]
	Aortic smooth muscle cells	Activation of GPER attenuates ROS and NADP/NADPH production and prevent arterial stiffening via cAMP-mediated downregulation of Nox4	[19]
	Aortic smooth muscle cells	GPER inhibits smooth muscle cell proliferation and migration; upregulation of GPER expression attenuates injury-mediated vascular hypertrophy	[90]
	Aorta	Protocatechuic accelerates re-endothelization of the endothelium and reduces angiogenesis and neointima formation in association with GPER activation	[13]
	Wire myography/ Carotid artery, aorta	GPER activation reduces blood pressure, promotes vasodilation, and blocks serotonin-induced intracellular Ca2+ increase and vascular tone potentially via ERK1/2	[14]
	Wire myography/ Carotid artery	GPER agonists elicit endothelium/NO-dependent relaxation; G1 scavenges superoxide	[36]
	Pressure myography/ Cerebral penetrating arterioles	GPER-induced relaxation is partially dependent on endothelial NO pathway; GPER exerts vasoprotective effect after hypoxia/reoxygenation injury	[37]
	Wire myography/ Mesenteric artery	GPER-activated relaxation is blunted in young males and aged females and is associated with a decrease in GPER protein expression	[38]
**Swine**	Wire myography/ Coronary artery smooth muscle cells	GPER activation involves cAMP/PKA signalling to induce relaxation via Epac/Rap1-mediated inhibition of RhoA/Rho kinase pathway	[91, 92]
	Wire myography/ Coronary artery	GPER activation relaxes coronary artery smooth muscle by increasing potassium efflux via BK(Ca) channels	[93]
	Wire myography/ Coronary artery smooth muscle cells	GPER activation causes relaxation via cAMP signalling and pre-incubation with G1 potentiates ET-1-precontraction through EGFR transactivation and ERK1/2 activation via Gβγ/Src/MMP pathway	[94]
	Aortic endothelial cells	GPER activation inhibits plasma membrane Ca(2+)-ATPase (PMCA) activity through tyrosine phosphorylation of the pump or by constitutive physical interaction between GPER and PMCA4b	[95]

	Organ chamber/ Epicardial coronary artery	GPER activation induces NO/endothelium-dependent vasodilation and attenuates ET-1 vasoconstriction	[96]
**Bovine**	Coronary artery endothelial cells	(-)-epicatechin activation of ERK 1/2 and CaMKII is dependent on the GPER/c-SRC/EGFR axis and GPER activation promotes NO production via eNOS phosphorylation	[97, 98]
**Rabbit**	Wire myography/ Basilar cerebral artery	Bazedoxifene-induced endothelium-independent relaxations via GPER involves Kv and L-type Ca2+ channels	[99]

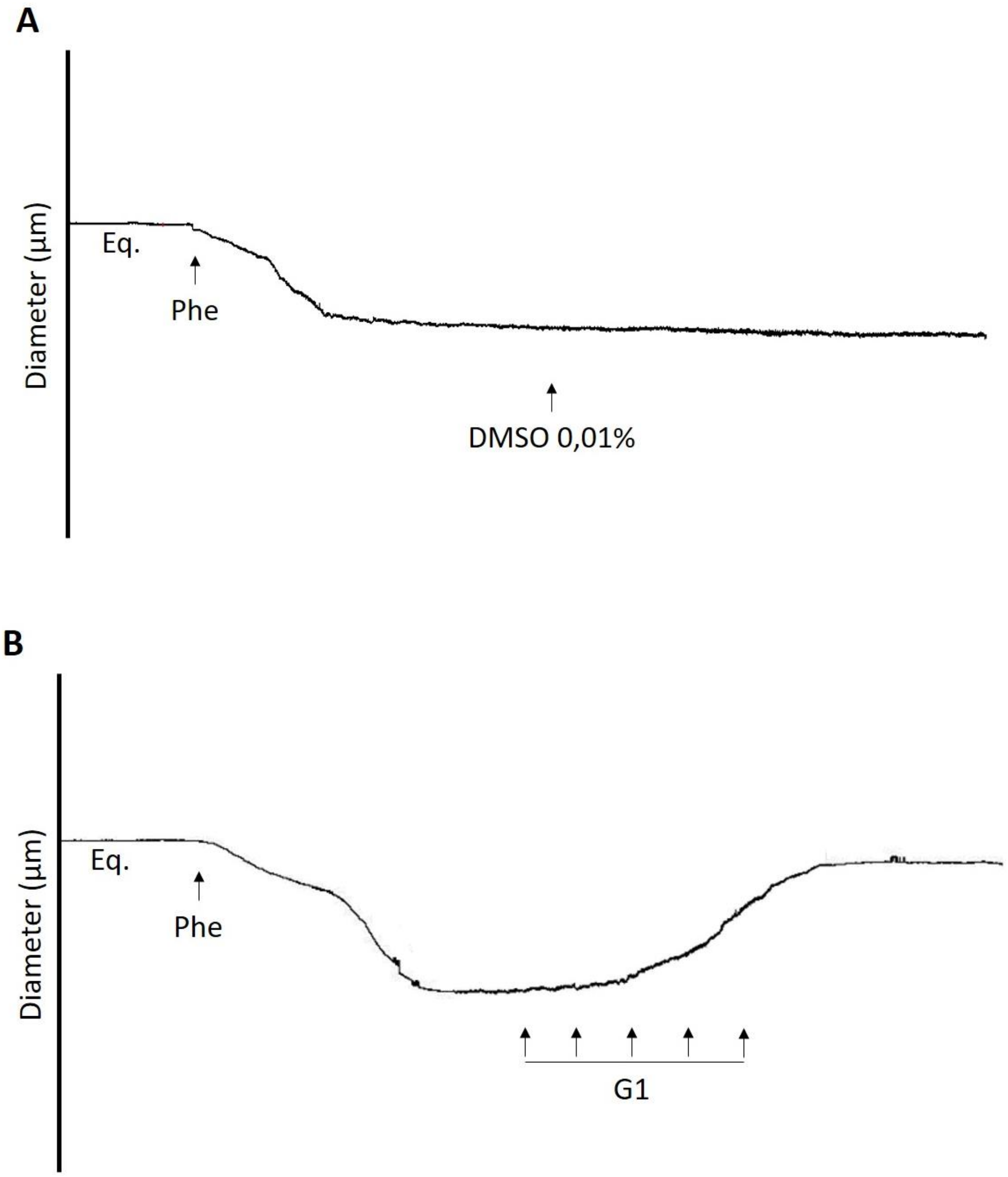

Figure S1. An example of representative experimental traces. The uterine arteries after equilibration (Eq.) are precontracted with Phenylephrine (Phe) and then were tested with DMSO (G1 vehicle, A) or with increase concentration of specific agonist of GPER, 1-(4-(-6-Bromobenzol(1,3) diodo5-yl)3a,4,5,9b-tetrahidro-3Hcyclopenta(c-)quinolin-8yl)ethanone (G1, B).
